# The Effect of Sowing Dates on Grain Yield and Quality in Spring Wheat (*Triticum aestivum* L.)

**DOI:** 10.1002/fsn3.70035

**Published:** 2025-05-05

**Authors:** Saeed Bagherikia, Habibollah Soughi, Manoochehr Khodarahmi, Fariba Naghipour

**Affiliations:** ^1^ Horticulture Crops Research Department Golestan Agricultural and Natural Resources Research and Education Center, AREEO Gorgan Iran; ^2^ Seed and Plant Improvement Institute AREEO Karaj Iran

**Keywords:** grain filling, grain quality, heat stress, protein, sowing dates

## Abstract

The impact of sowing dates on wheat ( *Triticum aestivum* L.) quality and yield was investigated comprehensively across two locations during the growing seasons from 2020 to 2022. The study revealed contrasting effects of temperature on grain protein content and glutenin composition. The increase in temperature during the grain‐filling period, caused by delayed sowing dates, led to an increase in grain protein content and wet gluten, while concurrently decreasing SDS and Zeleny sedimentation volumes, indicative of reduced gluten strength. This opposing trend underscores the complex relationship between temperature and wheat grain quality, influenced by the synthesis and polymerization of glutenin proteins critical for dough elasticity and baking performance. Furthermore, a negative relationship between ambient temperature and grain yield was observed across locations and years, highlighting the detrimental impact of heat stress on wheat productivity. Early sowing dates, which extended the grain‐filling period under cooler conditions, favored higher grain yield and superior gluten quality, characterized by higher SDS and Zeleny values. In contrast, delayed sowing dates, exposing wheat to higher temperatures during grain filling, resulted in increased grain protein content and wet gluten but compromised gluten strength. The findings underscore the importance of optimizing sowing dates and developing heat‐resilient wheat varieties to mitigate the adverse effects of climate change on wheat production.

## Graphical abstract

The study investigated the effects of sowing dates on wheat ( *Triticum aestivum* L.) yield and quality. Delayed sowing increased grain protein content and wet gluten but reduced gluten strength (SDS and Zeleny volumes) due to elevated temperatures during grain filling. Early sowing, under cooler conditions, enhanced grain yield and gluten quality. The findings highlight the importance of optimizing sowing dates and breeding heat‐resilient cultivars to mitigate heat stress and maintain wheat productivity under changing climatic conditions.

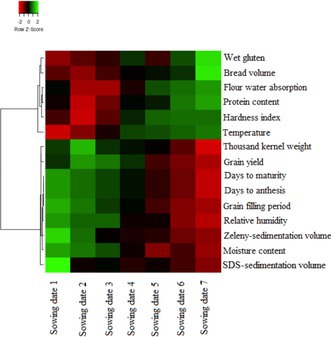

## 1 Introduction

Wheat ( *Triticum aestivum* L.) is one of the most important cereal crops worldwide, serving as a staple food for a significant portion of the global population (Shewry and Hey 2015). In Iran, wheat is the most important agricultural product concerning production and area under cultivation, which is very important in terms of both economy and food security (Ghaffari and Jalal Kamali 2013). Climate change is well known as one of the most important research challenges for plant scientists. Increased greenhouse gases and the effects of carbon dioxide have changed the temperature (TEMP) and rainfall pattern (IPCC 2018) resulting in reduced yield of many crops including wheat (Asseng et al. 2013). In many parts of the world, including Iran, the occurrence of terminal heat and drought stresses has been the main causes of decrease in wheat yield (Farooq et al. 2011). The sowing date influences grain yield (YLD) and quality by determining the thermal conditions during the grain‐filling period. The delayed sowing date causes the grain‐filling period to overlap with higher temperatures, thereby reducing the length of the grain‐filling period (Ahmed and Hassan 2015). The grain's protein content (PROT) and gluten quality are key determinants of its suitability for various end uses, particularly in baking, where the strength and elasticity of gluten are crucial for dough formation and bread quality (Irmak et al. 2008; Wang et al. 2020). Heat stress is considered more damaging to plants than other abiotic stresses, such as salinity, drought, and nutrient imbalances (Ashraf 2014). Environmental conditions, especially TEMP, during the grain‐filling period have a profound impact on wheat yield and quality (Zampieri et al. 2017). As global temperatures rise, understanding how heat stress affects wheat grain composition becomes increasingly important for developing strategies to mitigate the adverse effects on crop production (Asseng et al. 2015). Elevated temperatures during the grain‐filling period can accelerate the maturation process, often leading to reduced grain size and yield (Farooq et al. 2011). However, the relationship between TEMP and grain quality attributes, such as PROT and gluten strength, is complex and requires thorough investigation (Zhong et al. 2022). Recent studies have shown that increased ambient temperatures during grain filling can enhance grain PROT but often at the cost of gluten quality (Nuttall et al. 2017; Kino et al. 2020). High temperatures are known to disrupt the normal synthesis and polymerization of glutenin proteins, which are essential for forming a strong gluten network, thus negatively impacting dough strength and baking performance (Skylas et al. 2005; Koga et al. 2016). The sowing date plays a critical role in determining wheat yield and quality (Ahmed and Hassan 2015). Determining the appropriate sowing date to employ the optimal conditions is an important tool to decrease the effects of terminal heat and drought stresses (Andarzian et al. 2015).

This study aimed to investigate the effects of location, year, sowing date, and cultivar on the YLD and quality properties of wheat, with a specific focus on grain PROT and glutenins across different sowing dates. By analyzing data from different environments and growing seasons, we seek to provide comprehensive insights into optimizing wheat production under varying climatic conditions.

## 2 Materials and Methods

### 2.1 Field Experiments

The experiment was conducted at two agricultural research stations in Gorgan and Gonbad, Golestan province, within the warm and humid agroclimatic zone of northern Iran, during the 2020–2021 and 2021–2022 growing seasons. Monthly climatic data for these two growing seasons, including accumulated day length, precipitation, actual sunshine hours, mean relative humidity, mean temperature, and maximum temperature, are presented in Table 1.

**TABLE 1:** Meteorological statistics in two agricultural research stations during two cropping seasons (2020–2022).

Month	Location	Accumulated day length (hour)	Precipitation (mm)		Accumulated sunny hours (hour)		Mean relative humidity (%)		Mean temperature (°C)		Mean maximum temperature (°C)	
		2020–2021	2020–2021	2021–2022	2020–2021	2021–2022	2020–2021	2021–2022	2020–2021	2021–2022	2020–2021	2021–2022
		2021–2022										
Nov	Gorgan^a^	314.4	24.4	41.7	174.5	176.8	71	71	15.9	12.5	22.4	18.2
	Gonbad^b^	312.7	16.5	23.5	192.9	182.4	66	68	16.5	13.4	23.0	19.6
Dec	Gorgan	293.0	37.2	18.6	92.5	173.7	81	72	8.1	12.0	12.7	18.9
	Gonbad	291.0	31.9	12.8	107.3	176.1	78	64	8.9	13.5	13.6	20.6
Jan	Gorgan	293.3	21.9	61.0	154.5	161.3	74	72	7.2	8.8	13.6	15.2
	Gonbad	291.4	31.8	36.9	173.5	168.0	72	68	8.3	10.1	14.7	16.1
Feb	Gorgan	315.2	25.0	101.2	165.0	193.3	74	73	9.4	8.1	16.2	14.6
	Gonbad	314.1	24.5	61.1	187.8	201.0	71	65	10.2	9.5	17.6	16.2
Mar	Gorgan	335.9	72.3	59.2	149.5	117.0	72	80	8.4	11.8	14.6	16.9
	Gonbad	335.7	62.2	70.9	163.3	126.5	73	76	9.1	12.1	15.5	17.4
Apr	Gorgan	395.3	16.8	24.0	187.5	154.9	71	76	16.3	14.4	23.6	20.9
	Gonbad	396.2	16.6	11.9	202.7	173.6	67	69	16.6	15.5	23.6	22.1
May	Gorgan	429.4	13.8	42.6	209.2	139.9	70	76	21.3	18.9	28.1	24.1
	Gonbad	431.2	20.0	87.6	240.1	147.5	62	74	22.3	19.9	29.6	25.7
Jun	Gorgan	451.3	9.4	1.2	247.4	323.3	66	61	27.1	25.7	34.3	33.8
	Gonbad	455.0	12.2	2.2	279.0	334.0	50	53	28.2	26.3	36.3	34.8

^a^ Gorgan: Latitude 36°54′ N; Longitude 54°25′ E; Altitude 6 m.

^b^ Gonb: Latitude 37°16′ N; Longitude 55°13′ E; Altitude 45 m.

### 2.2 Plant Materials and Experimental Design

The plant materials consisted of the latest wheat cultivars released for northern Iran (South Caspian Sea basin), specifically four spring bread wheat cultivars: Arman, Araz, Taktaz, and N‐93‐9. The pedigrees of these cultivars are detailed in Table 2. The experimental design was a split‐plot within a randomized complete block design (RCBD) with three replications. The main plots included seven sowing dates (SD1: November 1, SD2: November 11, SD3: November 21, SD4: December 1, SD5: December 11, SD6: December 21, and SD7: December 31), whereas the subplots included the four bread wheat cultivars (Arman, Araz, Taktaz, and N‐93‐9). Irrigation was scheduled based on plant water requirements, with the first irrigation applied immediately after sowing. Sowing was performed using an experimental plot seeder (Wintersteiger, Ried, Austria), and each plot had a harvesting area of 7.2 m^2^. Seed density was set at 350 seeds per m^2^.

**TABLE 2:** Pedigree and release year of four bread wheat cultivars used in this study.

Cultivar	Year of release	Pedigree
Araz	2021	VOROBE
Arman	2021	PBW343/TONI//TROST/3/SOVA
N‐93‐9	—	CHAPIO/3/BORL95/2*EXCALIBUR//EXCALIBUR
Taktaz	2021	SAUAL/3/MILAN/S87230//BAV92

### 2.3 Crop Protection, Phenological Development, and Harvesting

Chemical fertilizers were applied based on the physical and chemical properties of the field soil (Table 3). Weed control was managed by applying a mixture of Granstar (20 g ha^−1^) and Topic (1‐l ha^−1^) herbicides. Agrotechnical practices were uniformly applied to all treatments throughout the growing period. Phenological stages, including emergence (ZGS 11), heading (ZGS 55), anthesis (ZGS 65), and physiological maturity (ZGS 90), were recorded for each cultivar according to Zadoks et al. (1974). At the end of each cropping season, harvesting was carried out using a small plot combine harvester (Wintersteiger, Austria), and YLD for each plot was measured using a digital scale.

**TABLE 3:** Soil physical and chemical characteristics at the two locations of the experiment.

Characteristic	Gorgan	Gonbad
Depth (cm)	0–30	0–30
Saturation percentage	43.6	43.6
Ec (dS/m)	1.7	0.6
PH	7.5	7.8
Organic matter (%)	1.6	1
Total *N* (%)	0.16	0.15
Available phosphorus (ppm)	9.6	13
Available potassium (ppm)	482	458
Clay (%)	30	35
Silt (%)	56	43
Sand (%)	14	22
Soil texture	Silty clay loam	Clay loam

### 2.4 Analysis of Quality Properties

Seed quality was assessed by analyzing several quality properties. Samples were prepared using a winnower machine (model: a/s Rationel Kornservice, Denmark) and then ground into flour using a hammer mill (Laboratory Mill 3100, Germany) and a roller mill (Brabender, Germany). PROT, water absorption (WA), bread volume (BV), and grain hardness index (HI) were measured using a NIR Analyzer (Perten Instruments, Sweden), following ICC standard no. 159 (ICC 1998). Wet gluten (WGLUT) was determined using a Gluten Analyzer (Perten Instruments, Sweden) as per AACC standard no. 38–11 (AACC 2000). Zeleny sedimentation volume (ZEL) was measured according to AACC standard no. 54–11, and sedimentation height (SDS) was measured as described by Carter et al. (1999). Thousand kernel weight (TKW) was determined using a grain counter and a precise scale with a sensitivity of 0.01 g.

### 2.5 Statistical Analysis

Analysis of variance and mean comparisons were conducted using SAS 9.4 software (SAS Inc., Cary, NC, USA) with the GLM procedure. Mean comparisons were performed using the LSD method (*p*‐value = 0.05). Cluster analysis and graphical representations were created using Online Heatmapper software (http://heatmapper.ca/expression/). Cluster analysis utilized Euclidean distance and the unweighted pair group method with arithmetic mean (UPGMA) method. The GGE Biplot software was utilized for the graphical presentation of the results (Yan 2001). Correlation analysis and simple linear regression analysis were performed using R software (R Core Team 2021) and Excel software, respectively.

## 3 Results

### 3.1 Grain Yield and Quality Properties

The results of the combined analysis of variance indicated that the main effects of sowing date and cultivar were statistically significant for all traits, including YLD, TKW, PROT, ZEL, baking value (BV), moisture content (MOIST), WA, HI, WGLUT, and sedimentation height (SDS) (Table 4). The main effect of location was significant for PROT, ZEL, BV, MOIST, WGLUT, and SDS traits. Moreover, the main effect of year was significant for all traits except PROT and WA. Although some interaction effects were significant for the traits studied, the interaction between sowing date × cultivar was significant for all traits except TKW (Table 4). In line with the study's objective, which aimed to explore the relationship between sowing dates (the associated temperature variations) and grain quality traits, the mean comparisons focused on the main effects.

**TABLE 4:** Combined analysis of variance (ANOVA) for grain yield and quality properties.

S.O.V	df	Mean squares									
		YLD	TKW	PROT	ZEL	BV	MOIST	WA	HI	WGLUT	SDS
Location (L)	1	8574^ns^	0.20^ns^	12.81**	231.7**	40656**	63.18**	0.45^ns^	16.3^ns^	209.32**	1586.0**
Year (Y)	1	25995667**	311.24*	0.01^ns^	2015.9**	98332**	165.62**	4.10^ns^	1517.3**	326.47**	434.3**
L × Y	1	359^ns^	161.65^ns^	22.22**	73.4*	10164^ns^	0.01^ns^	50.69**	304.8**	648.52**	123.9^ns^
Erorr1	8	291,818	33.60	0.25	8.4	2098	1.86	1.04	6.2	12.61	47.9
Sowing date (SD)	6	14069865**	68.59*	1.52*	89.3**	2932*	1.78**	4.18**	43.9**	38.34*	251.9**
L × SD	6	2791321**	13.13^ns^	0.99^ns^	14.4^ns^	1919^ns^	0.50^ns^	1.10^ns^	9.5**	6.32^ns^	469.8**
Y × SD	6	1436910^ns^	50.87^ns^	0.47^ns^	17.9^ns^	1931^ns^	0.40^ns^	1.63^ns^	9.4**	8.58^ns^	167.7^ns^
L × Y × SD	6	547404^ns^	19.44^ns^	1.38*	34.2**	1075^ns^	1.45**	1.83^ns^	26.0^ns^	6.32^ns^	77.7^ns^
Erorr2	48	635,616	28.25	0.53	10.3	1121	0.35	1.06	2.2	16.64	73.4
Cultivar (C)	3	1009474*	1054.71**	17.07**	59.4**	197821**	15.90**	169.90**	1140.7**	554.91**	313.5**
SD × C	18	523904*	25.75^ns^	0.18*	15.0**	1039**	0.14*	1.08*	4.1*	6.31**	130.0**
Y × C	3	2011691**	33.35^ns^	0.14^ns^	31.2**	1037^ns^	0.19^ns^	1.24^ns^	5.8^ns^	7.75^ns^	224.01**
L × C	3	399710^ns^	22.81^ns^	0.21^ns^	7.2^ns^	1075^ns^	0.10^ns^	2.28*	6.1^ns^	5.51^ns^	120.6^ns^
L × Y × C	3	1748572**	14.21^ns^	0.15^ns^	7.8^ns^	667^ns^	0.01^ns^	0.61^ns^	1.9^ns^	5.51^ns^	23.8^ns^
Y × SD × C	18	123078^ns^	22.63^ns^	0.26**	6.2^ns^	674^ns^	0.08^ns^	0.83^ns^	2.8^ns^	3.38^ns^	81.7^ns^
L × SD × C	18	233756^ns^	21.12^ns^	0.13^ns^	6.4^ns^	693^ns^	0.12^ns^	0.92^ns^	3.6^ns^	0.92^ns^	46.6^ns^
L × Y × SD × C	18	289740^ns^	22.13^ns^	0.14^ns^	6.4^ns^	619^ns^	0.11^ns^	0.74^ns^	1.7^ns^	0.92^ns^	45.4^ns^
Erorr3	168	272,092	19.40	0.11	4.1	439	0.08	0.64	2.4	2.99	50.7
CV%		8.28	9.80	2.55	7.08	4.11	3.44	1.24	3.11	5.23	8.87

Abbreviations: BV, bread volume; HI, hardness index; MOIST, moisture content; ns, nonsignificant; PROT, protein; TKW, thousand kernel weight; WA, flour water absorption; WGLUT, wet gluten and SDS, sedimentation height; YLD, grain yield; ZEL, Zeleny sedimentation volume.

* and ** are significant at 0.05 and 0.01 probability levels, respectively.

Table 5 presents the results of mean comparisons for the main effects of location, year, sowing date, and cultivar on YLD and quality properties. There was no statistically significant difference between the two locations in terms of YLD and TKW. PROT, BV, and WGLUT were higher in Gonbad compared with Gorgan, whereas ZEL, MOIST, and SDS showed higher values in Gorgan. Other quality properties did not differ significantly between the two locations. YLD, TKW, ZEL, MOIST, and SDS were significantly higher in the second year compared with the first year (Table 5). Conversely, BV, HI, and WGLUT were higher in the first year than in the second year. There was no significant difference in PROT between the 2 years of the experiment (Table 5).

**TABLE 5:** Mean comparison for effect of location, year, sowing date, and cultivar on grain yield and quality properties.

S.O.V	YLD (kg ha^−1^)	TKW (g)	PROT (%)	ZEL (mL)	BV (mL)	MOIST (%)	WA (%)	HI	WGLUT (%)	SDS (mm)
Location										
Gorgan	5454.9a	38.88a	12.70b	29.64a	498.27b	8.87a	64.52a	49.84a	32.26b	82.47a
Gonbad	5424.3a	38.75a	13.05a	27.80b	520.27a	8.00b	64.59a	50.28a	33.92a	78.63b
Year										
2020–2021	5182.7b	38.22b	12.90a	26.18b	526.38a	7.73b	64.66a	52.18a	34.11a	79.16b
2021–2022	5690.0a	39.36a	12.85a	31.08a	492.17b	9.14a	64.44a	47.93b	32.06b	81.93a
Sowing date										
SD1^§^	5618.4b	39.29ab	12.86abc	31.00a	505.58ab	8.69a	64.56ab	49.56c	32.38b	85.38a
SD2	6051.7a	40.23a	12.60c	29.83ab	503.23b	8.62ab	64.18b	48.46d	32.63ab	80.31b
SD3	5947.0a	39.17ab	12.72bc	28.54bc	506.58ab	8.56abc	64.17b	49.38c	32.85ab	80.63b
SD4	5612.6b	38.96ab	12.89abc	28.31 cd	509.10ab	8.41bcd	64.50a	50.31b	33.33ab	79.85b
SD5	5159.6c	38.77abc	12.91ab	28.23 cd	509.96ab	8.23d	64.74a	50.83ab	32.79ab	80.25b
SD6	4949.4c	38.15bc	13.05a	27.46 cd	511.40ab	8.33 cd	64.82a	50.94a	33.46ab	79.33b
SD7	4711.3d	37.20c	13.11a	27.06d	519.06a	8.20d	64.91a	50.94a	34.17a	78.08b
Cultivar										
Araz	5530.0a	42.29a	12.27d	28.81a	580.45a	9.09a	62.45c	44.64d	33.81b	82.52a
Arman	5466.6a	37.17c	13.02b	27.40b	478.62c	8.17b	65.40a	51.92b	33.43b	77.98b
N‐93‐9	5275.3b	35.20d	12.86c	29.27a	499.92b	8.25b	64.90b	50.94c	29.89c	81.35a
Taktaz	5490.3a	40.59b	13.35a	29.05a	478.11c	8.24b	65.47a	52.74a	35.21a	80.35a

*Note:* Means followed by at least one letter in common are not significant different (LSD = 0.05).

Abbreviations: BV, bread volume; HI, hardness index; MOIST, moisture content; PROT, protein; SDS, sedimentation height; TKW, thousand kernel weight; WA, flour water absorption; WGLUT, wet gluten; YLD, grain yield; ZEL, Zeleny sedimentation volume.

^§^ SD1 (November 1), SD2 (November 11), SD3 (November 21), SD4 (December 1), SD5 (December 11), SD6 (December 21), and SD7 (December 31).

The highest YLD was observed with sowing dates SD2 (6052 kg/ha) and SD3 (5947 kg/ha), significantly surpassing other sowing dates (SD1, SD4, SD5, SD6, and SD7) (Table 5). Additionally, PROT, BV, WA, HI, and WGLUT were higher with later sowing dates compared to earlier ones, whereas ZEL and SDS showed higher values with earlier sowing dates (Table 5).

Mean comparisons for YLD showed that Araz (5530 kg/ha), Taktaz (5490 kg/ha), Arman (5466 kg/ha), and N‐93‐9 line (5275 kg/ha) had the highest performance, respectively. The YLD of N‐93‐9 line was significantly lower than other genotypes (Table 5). Araz had the highest TKW (42.29 g), whereas N‐93‐9 line had the lowest (35.20 g). Taktaz exhibited the highest PROT (13.35%), followed by Arman (13.02%), N‐93‐9 (12.86%), and Araz (12.27%). Taktaz also showed superior values in WA, HI, and WGLUT compared to other genotypes.

### 3.2 Length of Anthesis, Maturity Stages, and Grain‐Filling Period

Physiological maturity and the grain‐filling period were longer in Gorgan than in Gonbad. Conversely, the mean temperature during the grain‐filling period was lower in Gorgan than in Gonbad (Table 6). In the second year, both the physiological maturity stage and the grain‐filling period were longer compared to the first year (Table 6). The difference of 2 months between SD1 and SD7 resulted in a reduction of 41 and 49 days in the length of anthesis and physiological maturity stages, respectively. Additionally, the duration of the grain‐filling period significantly decreased by 7.8 days. Taktaz was the earliest cultivar, yet it also exhibited the longest grain‐filling period (Table 6).

**TABLE 6:** Mean comparison for effect of location, year, sowing date, and cultivar on length of phenological stages, mean temperature, and relative humidity during the grain‐filling period.

S.O.V	DAN (day)	DMA (day)	GFP (day)	TEMP (C°)	RH (%)
Location					
Gorgan	133.27a	171.30a	38.03a	19.64b	72.47a
Gonbad	132.55b	167.76b	35.21b	20.47a	68.35b
Year					
2020–2021	133.87a	167.08b	33.21b	20.74a	68.45b
2021–2022	131.95b	171.99a	40.04a	19.37b	72.36a
Sowing date					
SD1^§^	150.78a	191.44a	40.66a	18.43 g	71.63a
SD2	146.09b	185.63b	39.53b	19.04f	71.19b
SD3	140.73c	178.63c	37.89c	19.89e	71.21b
SD4	135.19d	172.02d	36.83d	20.57d	70.26c
SD5	127.77e	162.80e	35.03e	20.67c	70.26c
SD6	120.02f	153.59f	33.58f	20.82b	69.33d
SD7	109.80 g	142.64g	32.84g	20.97a	68.97e
Cultivar					
Araz	132.84c	169.40b	36.56b	20.05b	70.38c
Arman	136.24a	172.67a	36.43b	20.58a	69.77d
N‐93‐9	133.26b	169.43b	36.17b	20.05b	70.60b
Taktaz	129.30d	166.63c	37.33a	19.53c	70.88a

*Note:* Means followed by at least one letter in common are not significant different (LSD = 0.05).

Abbreviations: DAN, days to anthesis; DMA, days to maturity; GFP, grain‐filling period; RH, relative humidity; TEMP, temperature.

^§^ SD1 (November 1), SD2 (November 11), SD3 (November 21), SD4 (December 1), SD5 (December 11), SD6 (December 21), and SD7 (December 31).

### 3.3 Temperature and Relative Humidity During Grain‐Filling Period

The mean temperature during the grain‐filling period was lower in Gorgan compared to Gonbad, and in the second year it was lower than in the first year. Conversely, relative humidity (RH) during the grain‐filling period was higher in Gorgan compared to Gonbad, and in the second year it was higher than in the first year (Table 6). Delayed sowing dates resulted in a significant increase in mean temperature and decrease in RH during the grain‐filling period compared to early sowing dates. The TEMP and RH differed by +2.54 and −2.66%, respectively, between the initial and final sowing dates. Taktaz, the earliest cultivar, had the lowest mean temperature (19.53°C) and highest RH (70.88%) during the grain‐filling period (Table 6).

### 3.4 Effect of Mean Temperature During Grain Filling on Grain Yield and Quality Properties

**FIGURE 1:**
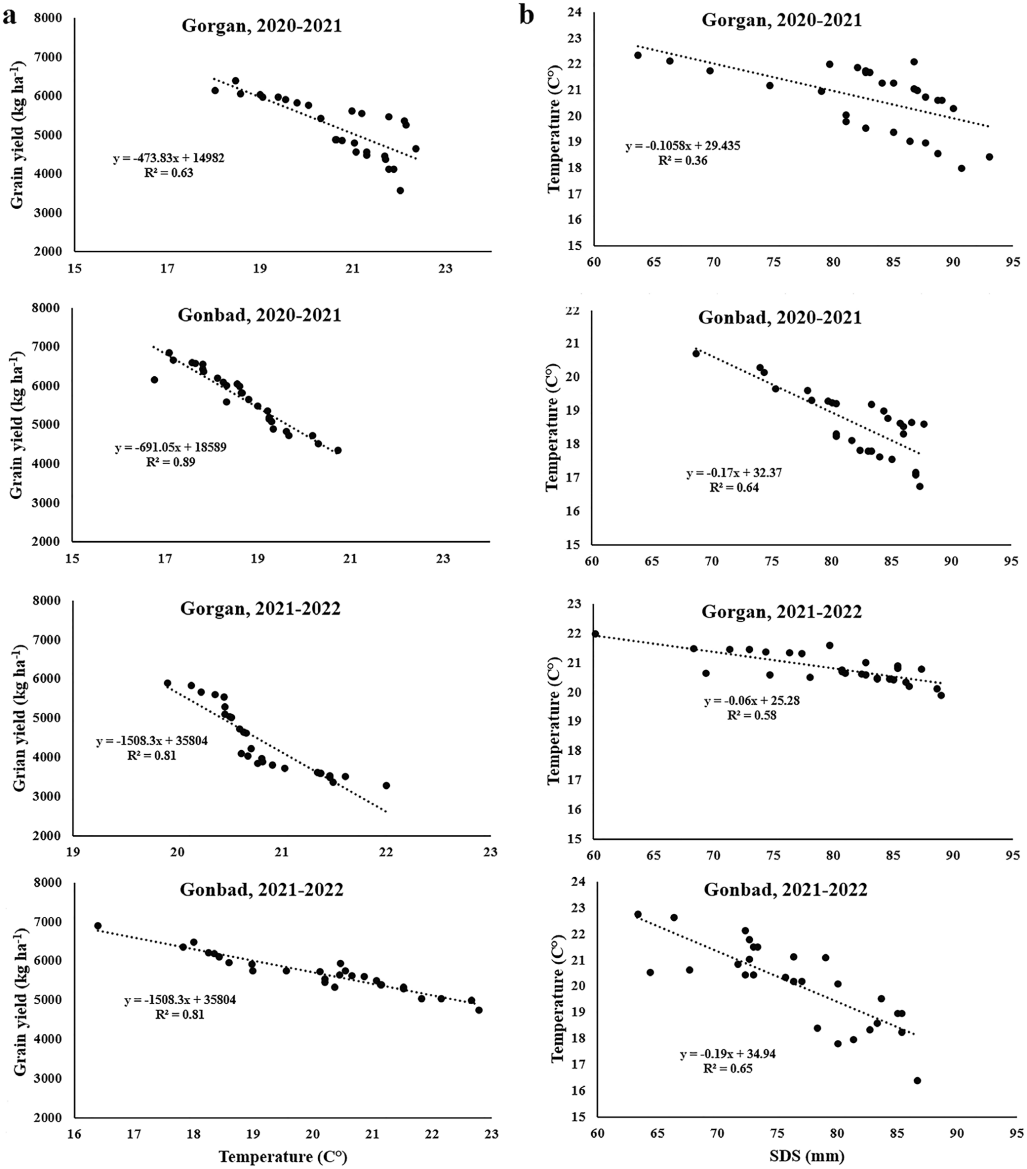
Relationship between mean temperature during grain filling with grain yield (a) and SDS (b). The temperature fluctuations during the grain‐filling period were a result of the different sowing dates.

**FIGURE 2:**
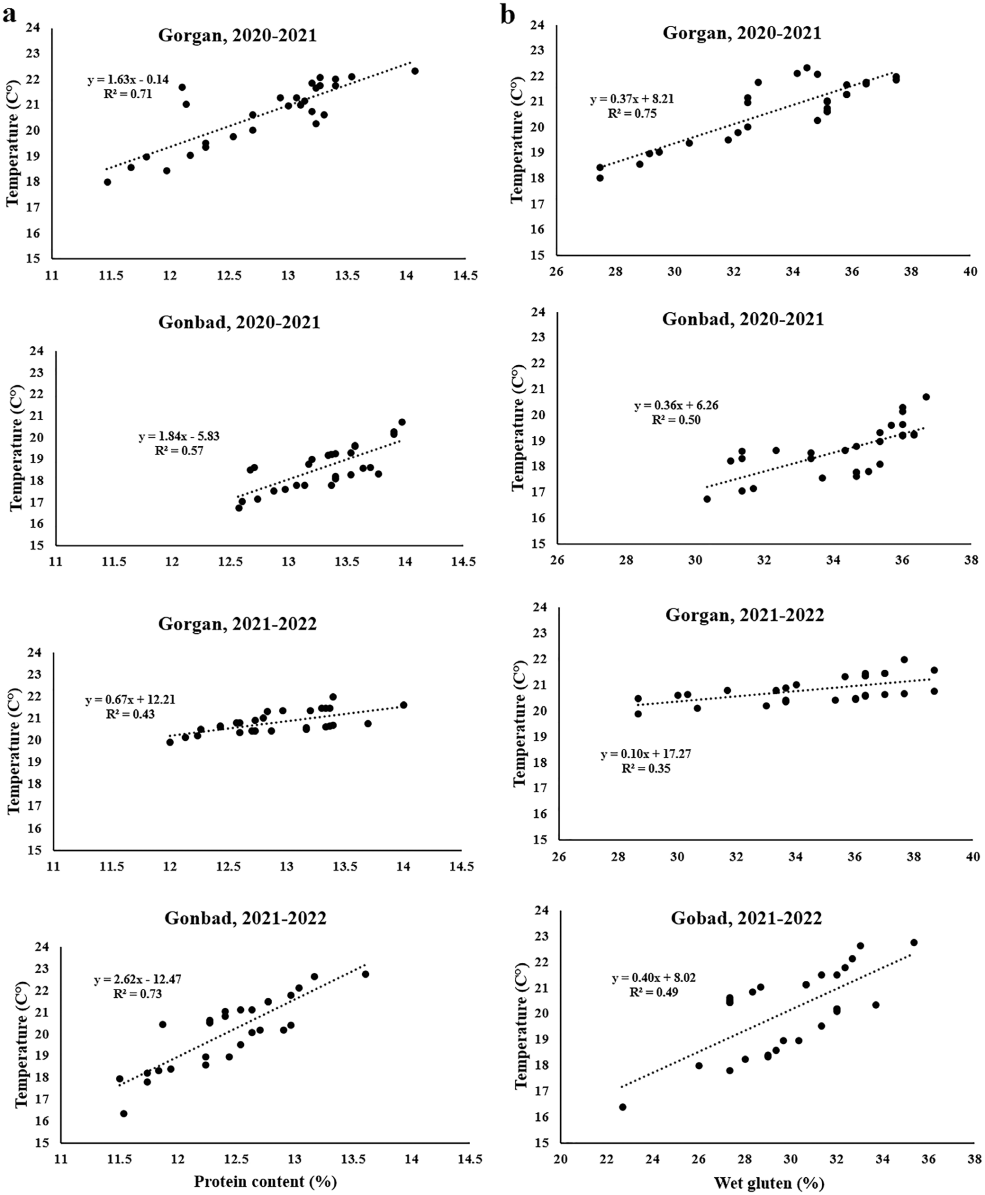
Relationship between mean temperature during grain‐filling with protein content (a) and wet gluten (b). The temperature fluctuations during the grain‐filling period were a result of the different sowing dates.

Simple linear regression analysis indicated a negative relationship between mean temperature during grain filling and YLD across all locations and years of the experiment (*R* ^2^ = 0.63–0.89; Figure 1a). Similarly, a negative relationship was observed between mean temperature during grain filling and SDS (*R* ^2^ = 0.36–0.65; Figure 1b). Conversely, a positive relationship existed between mean temperature during grain filling and PROT (*R* ^2^ = 0.43–0.73; Figure 2a) as well as WGLUT (*R* ^2^ = 0.35–0.75; Figure 2b). These results indicate that an increase in temperature during delayed sowing dates led to decreased YLD and SDS but increased PROT and WGLUT.

### 3.5 Relationship of Protein Content With Grain Yield and Grain‐Filling Period

**FIGURE 3:**
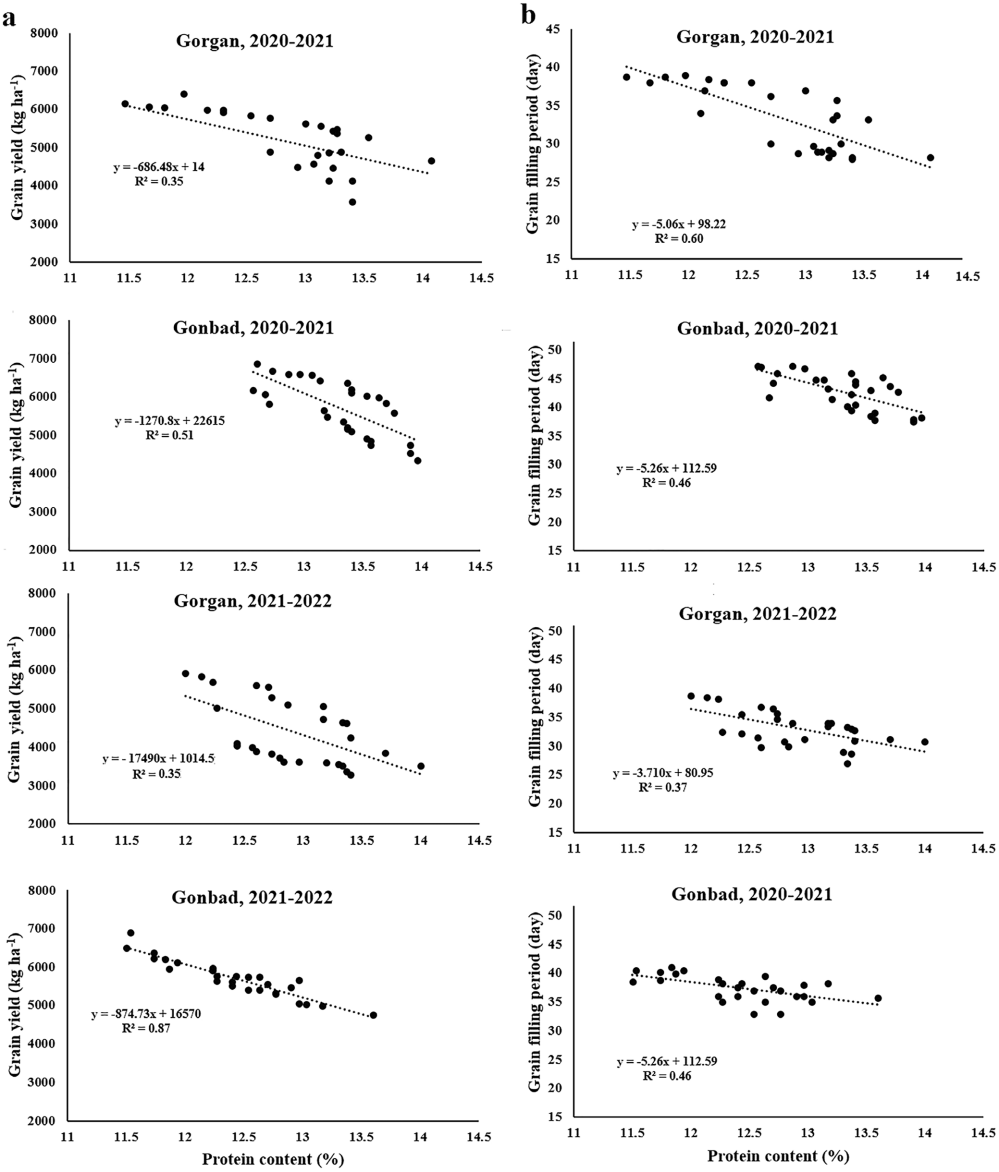
Relationship between protein content with grain yield (a) and grain‐filling period (b).

Linear regression analysis revealed a negative relationship between PROT and YLD across all locations and years of the experiment (*R* ^2^ = 0.35–0.87; Figure 3a). Similarly, a negative relationship was observed between PROT and grain‐filling period (*R* ^2^ = 0.37–0.60; Figure 3b). These findings suggest that in delayed sowing dates, where the grain‐filling period is shortened due to terminal heat stress, YLD decreases while PROT increases.

### 3.6 Clustering Heatmap and Principal Component Analysis

**FIGURE 4:**
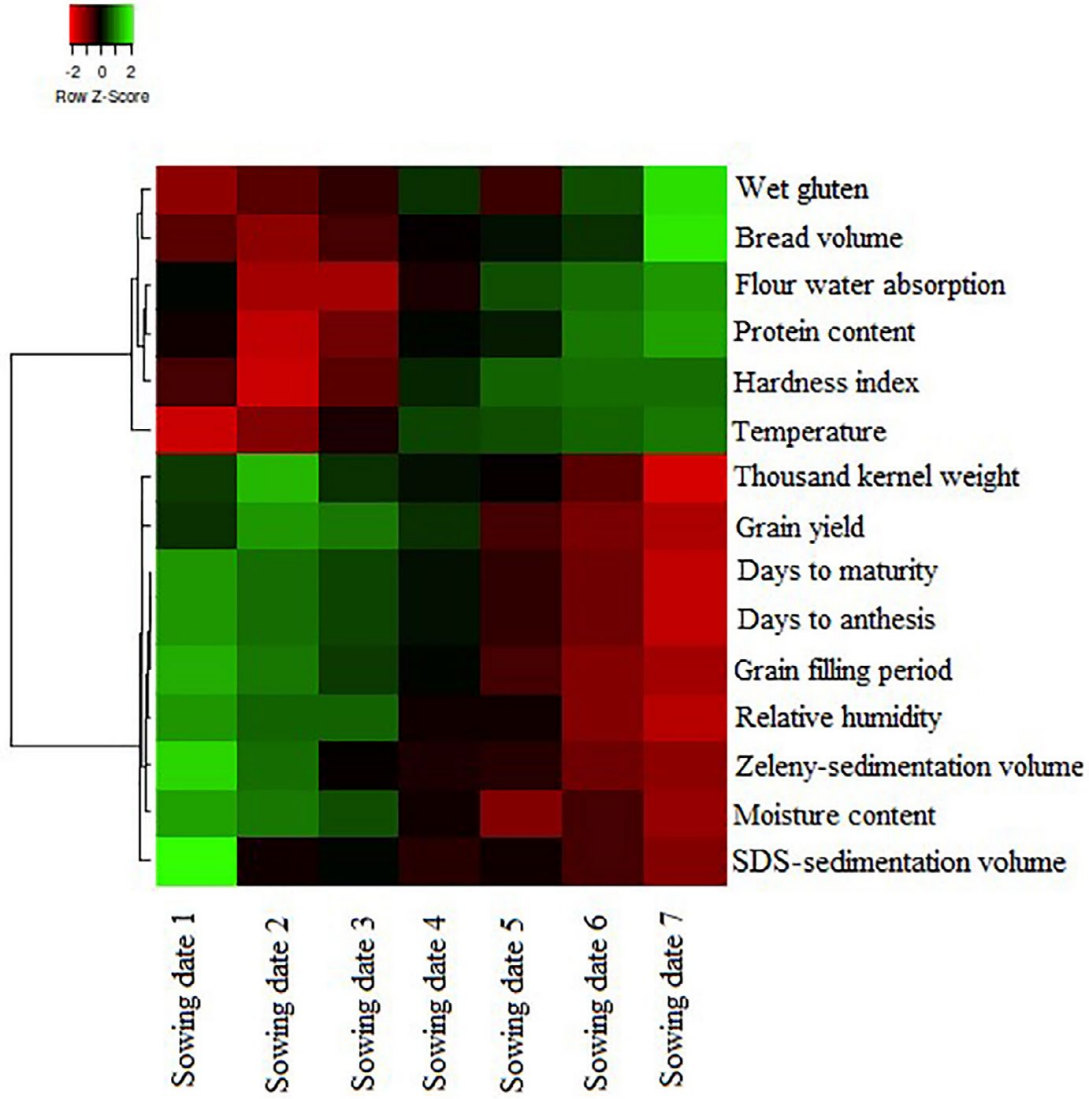
Cluster analysis and graphical display of the baking quality traits. The darker green indicates a higher value, conversely the darker red indicates a lower value. The black indicates the mean value of the parameter that is equal to the total mean.

Cluster analysis categorized quantitative and qualitative traits under different sowing dates (Figure 4). Traits such as WGLUT, BV, WA, PROT, HI, and TEMP formed one group, whereas traits like TKW, YLD, days to anthesis (DAN), days to physiological maturity (DMA), RH, ZEL, MOIST, and SDS formed another group. The heatmap indicated that traits in the first group had lower values in the early sowing dates (SD1, SD2, SD3) and higher values in the late sowing dates (SD5, SD6, SD7). Conversely, traits in the second group showed higher values in the early sowing dates and lower values in the late sowing dates, with SD4 exhibiting intermediate values for both groups.

**FIGURE 5:**
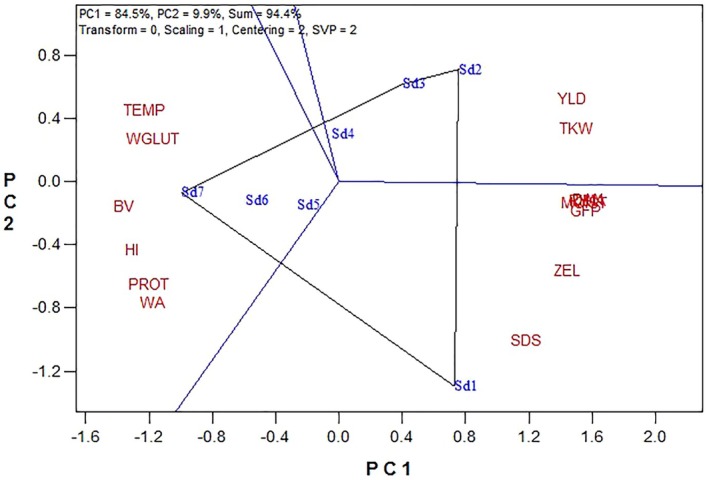
A polygon view of the GGE biplot shows the “which is best for what”, based on baking quality traits. BV, bread volume; DAN, days to anthesis; DMA, days to maturity; GFP, grain‐filling period; HI, hardness index; MOIST, moisture content; PROT, protein; TEMP, temperature and RH, relative humidity; TKW, thousand kernel weight; WA, flour water absorption; WGLUT, wet gluten and SDS, sedimentation height; YLD, grain yield; ZEL, Zeleny sedimentation volume. SD1 (November 1), SD2 (November 11), SD3 (November 21), SD4 (December 1), SD5 (December 11), SD6 (December 21), and SD7 (December 31).

Principal component analysis (PCA) explained approximately 94% of the data variance (Figure 5), with traits similarly grouped as in cluster analysis. The polygon plot highlighted optimal sowing dates for each trait: SD1 and SD2 were favorable for YLD, TKW, DMA, DAN, GFP, ZEL, and SDS, whereas SD6 and SD7 were optimal for TEMP, WGLUT, BV, HI, PROT, and WA (Figure 5).

### 3.7 Correlation Analysis

**FIGURE 6:**
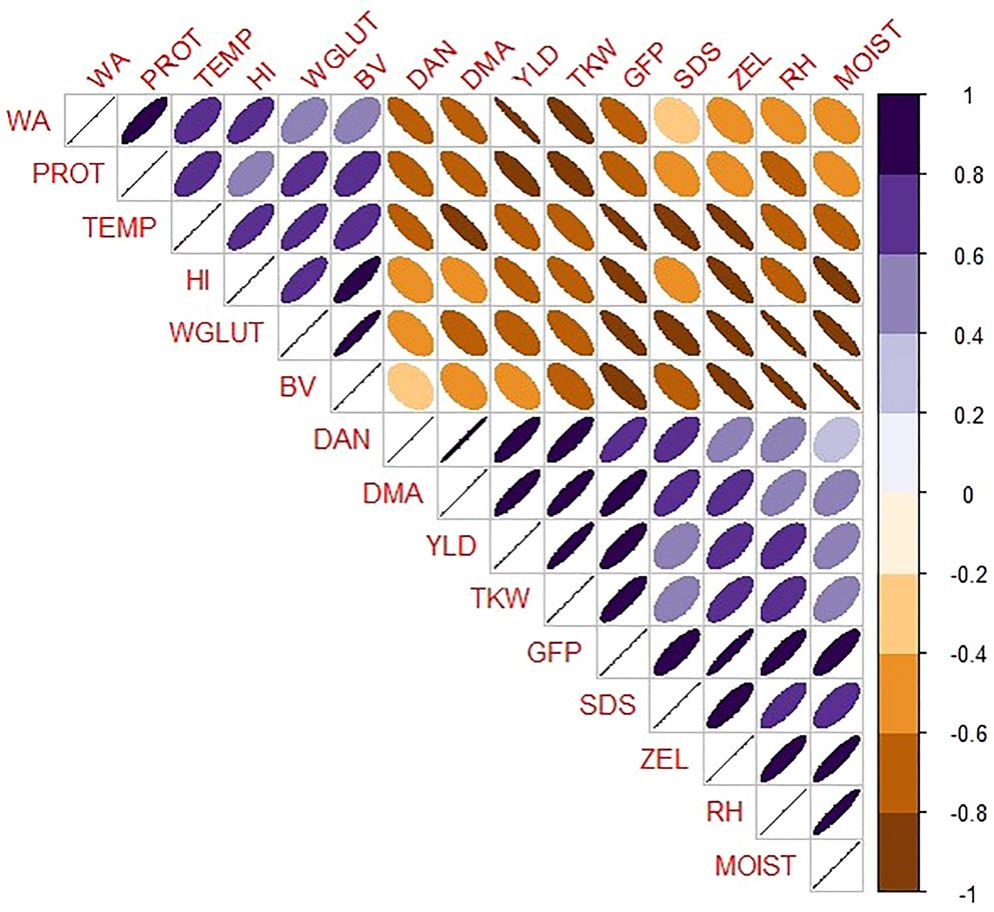
Pearson correlation between different baking quality traits in bread wheat cultivars. BV, bread volume; DAN, days to anthesis; DMA, days to maturity; GFP, grain‐filling period; HI, hardness index; MOIST, moisture content; PROT, protein; RH, relative humidity; SDS, sedimentation height; TEMP, temperature; TKW, thousand kernel weight; WA, flour water absorption; WGLUT, wet gluten; YLD, grain yield; ZEL, Zeleny sedimentation volume.

Pearson correlation analysis (Figure 6) revealed significant positive correlations between TEMP and PROT, WA, HI, WGLUT, and BV. Conversely, negative correlations were observed between TEMP and other quantitative and quality properties (DAN, DMA, YLD, TKW, GFP, SDS, ZEL, RH, MOIST). PROT showed significant positive correlations with WA, HI, WGLUT, and BV, and negative correlations with SDS, ZEL, and MOIST. YLD exhibited negative correlations with WA, PROT, HI, and WGLUT while showing positive correlations with SDS, ZEL, and MOIST. DAN, DMA, and GFP displayed negative correlations with PROT, but positive correlations with SDS and ZEL.

## 4 Discussion

The relationship between TEMP during the grain‐filling period and YLD and quality in wheat production is a complex interplay influenced by various environmental factors, agricultural practices, and genetic traits (Mutwali et al. 2016). The elevated temperatures during the grain‐filling period, which were clearly a result of delayed sowing dates for different wheat cultivars (Table 6), emphasize the complex impact of ambient TEMP on the qualitative and quantitative traits of wheat. One of the most compelling findings is the opposing trends in grain PROT and glutenins in response to increasing temperatures. Specifically, grain PROT and WGLUT increase with rising temperatures during grain filling, whereas SDS and ZEL decrease (Table 5). SDS and ZEL tests provide an indication of the gluten strength, which is influenced by the quality of glutenin proteins in wheat. Glutenin proteins are responsible for the elasticity and strength of gluten, which are crucial for determining dough quality and baking performance (Shewry and Tatham 1997).

Our finding showed a positive relationship existed between mean temperature during grain filling and PROT (Figure 2a) as well as WGLUT (Figure 2b) across all locations and years of the experiment. TEMP plays a critical role in determining the composition and quality of wheat grains (Ashraf 2014). Elevated temperatures during this period are associated with increased grain PROT, a phenomenon that aligns with findings from recent studies. For instance, heat stress has been shown to enhance the accumulation of nitrogenous compounds in grains, thereby increasing PROT (Nuttall et al. 2017; Kino et al. 2020). This effect can be attributed to the accelerated rate of protein synthesis driven by higher enzyme activity and enhanced metabolic rates under elevated temperatures (Ahmed and Hassan 2015; Zhong et al. 2022).

However, the increase in PROT does not necessarily translate to improved gluten quality. The reduction in SDS and ZEL values observed under higher temperatures (Figure 1b) indicates a decline in gluten strength and elasticity. This discrepancy can be explained by the differential impact of heat on glutenin and gliadin synthesis. High temperatures tend to disrupt the normal polymerization of glutenin subunits, which are crucial for forming a strong gluten network (Koga et al. 2016). Furthermore, heat stress usually increases gliadin synthesis, which reduces the glutenin/gliadin ratio and detrimentally affects flour quality (Bencze et al. 2004). Therefore, despite the higher PROT, the quality of the gluten deteriorates, as evidenced by lower SDS and ZEL values in the late sowing dates (Table 5).

The negative relationship between YLD and ambient temperature during grain‐filling period (Figures 1a and 6) is consistent with well‐documented adverse effects of heat stress on wheat yield (Asseng et al. 2015; Zampieri et al. 2017). High temperatures shorten the grain‐filling period (Table 6), reducing the duration for starch accumulation and leading to lower YLD and TKW (Zhang et al. 2012; Ullah et al. 2022). The decrease in TKW (Table 5) and GFP (Table 6) under delayed sowing dates, which expose plants to higher temperatures, further supports this observation. Interestingly, although higher temperatures decrease YLD (Figure 1a), they simultaneously increase grain PROT and WGLUT (Figure 2a,b). This inverse relationship between yield and PROT is a common phenomenon in cereal crops, often referred to as the dilution effect (Oury and Godin 2007). Essentially, when YLD is high, the relative concentration of protein decreases due to the larger amount of starch being synthesized. Conversely, under heat stress conditions that limit yield, the concentration of protein in the grain increases.

The study's examination of different sowing dates provides further insights into the TEMP effects during grain filling. Earlier sowing dates, which result in longer grain‐filling periods and lower temperatures, favor higher YLD and better gluten quality, as indicated by higher SDS and ZEL values (Table 5). Conversely, delayed sowing dates, which expose wheat to higher temperatures, result in higher grain PROT and WGLUT but lower SDS and ZEL values (Table 5). These results are consistent with other studies indicating that optimal sowing dates can mitigate the adverse effects of heat stress on wheat quality and yield (Farooq et al. 2011; Ahmed and Hassan 2015). The clustering analysis (Figure 4) and PCA (Figure 5) further illustrate that traits associated with high yield and quality (e.g., TKW, SDS, ZEL) cluster with early sowing dates, whereas traits linked to higher PROT and WGLUT cluster with late sowing dates. These analyses highlight the importance of selecting appropriate sowing dates to optimize both yield and quality under varying climatic conditions. New cultivars are often assumed to share the same optimal sowing dates as older ones, which is not always accurate. Therefore, conducting planting date experiments is crucial to determine the optimal sowing date for each cultivar and to provide farmers with precise guidelines. Moreover, although there are optimal sowing dates for wheat to maximize performance, several practical constraints, including climatic conditions, soil moisture conditions, pest control practices, and crop rotation schedules, often influence the actual sowing dates. These factors may cause farmers to delay sowing, potentially impacting the crop performance (Qiao et al. 2023). The contrasting effects of sowing dates on grain PROT and glutenins can be attributed to specific physiological and biochemical mechanisms. High temperatures in delayed sowing dates accelerate the grain‐filling process, which limits the time available for starch synthesis and accumulation. This accelerated development can lead to a higher proportion of nitrogenous compounds, thereby increasing PROT (Leghari et al. 2016). Moreover, heat stress affects the synthesis and assembly of gluten proteins. Glutenins, which are responsible for dough strength and elasticity, are particularly sensitive to high temperatures. Heat stress can disrupt the polymerization of glutenin subunits, leading to a weaker gluten network and thus lower SDS and ZEL values (Skylas et al. 2005; Irmak et al. 2008). This is supported by the negative correlation between TEMP and SDS/ZEL observed in this study (Figure 6).

The findings of this study have significant implications for wheat cultivation and breeding, particularly in the context of global climate change. As global temperatures rise, the frequency and intensity of heat stress events during critical growth periods are expected to increase, posing challenges for maintaining YLD and quality (Lobell et al. 2011; Asseng et al. 2019).

Breeding programs need to focus on developing wheat varieties that are resilient to heat stress. This includes selecting for traits such as extended grain‐filling periods, efficient nitrogen use, and stable gluten protein composition under elevated temperatures (Semenov and Halford 2009; Pinto et al. 2010). Agronomic practices, such as optimizing sowing dates, can also help mitigate the adverse effects of heat stress. Early sowing can extend the grain‐filling period under cooler conditions, enhancing both yield and quality. However, this must be balanced with the risk of frost damage during early developmental stages (Farooq et al. 2011).

## 5 Conclusion

This study elucidates the complex effects of increasing ambient temperature during the wheat grain‐filling period on grain PROT, glutenins, and overall grain quality. Higher temperatures boost grain PROT and WGLUT but negatively impact YLD and gluten quality, as measured by SDS and ZEL. These findings underscore the need for integrated strategies combining breeding, agronomy, and management practices to adapt wheat production to the challenges posed by climate change. Future research should focus on understanding the molecular and physiological mechanisms underlying these temperature effects, which will be crucial for developing resilient wheat cultivars and sustainable cultivation practices.

## Author Contributions

**Saeed Bagherikia:** conceptualization (lead), data curation (lead), formal analysis (lead), funding acquisition (lead), investigation (lead), project administration (equal), software (lead), validation (lead), visualization (lead), writing – original draft (lead), writing – review and editing (equal). **Habibollah Soughi:** formal analysis (supporting), investigation (supporting), methodology (supporting), software (supporting), supervision (supporting), writing – review and editing (equal). **Manoochehr Khodarahmi:** formal analysis (supporting), methodology (supporting), resources (equal), writing – review and editing (equal). **Fariba Naghipour:** data curation (supporting), investigation (supporting), methodology (supporting), validation (supporting), writing – review and editing (supporting).

## Ethics Statement

This study does not involve any human or animal testing.

## Conflicts of Interest

The authors declare no conflicts of interest.

## Data Availability

The data that support the findings of this study are available from the corresponding author upon reasonable request.

## References

[fsn370035-bib-0001] AACC . 2000. Approved Methods of the American Association of Cereal Chemists. Vol. 2. 10th ed, 877. American Association of Cereal Chemists.

[fsn370035-bib-0002] Ahmed, M. , and F. Hassan . 2015. “Response of Spring Wheat (*Triticum aestivum* L.) Quality Traits and Yield to Sowing Date.” PLoS One 10, no. 4: e0126097. 25927839 10.1371/journal.pone.0126097 PMC4415767

[fsn370035-bib-0003] Andarzian, B. , G. Hoogenboom , M. Bannayan , M. Shirali , and B. Andarzian . 2015. “Determining Optimum Sowing Date of Wheat Using CSM‐CERES‐Wheat Model.” Journal of the Saudi Society of Agricultural Sciences 14: 189–199.

[fsn370035-bib-0004] Ashraf, M. 2014. “Stress‐Induced Changes in Wheat Grain Composition and Quality.” Critical Reviews in Food Science and Nutrition 54, no. 12: 1576–1583. 24580559 10.1080/10408398.2011.644354

[fsn370035-bib-0006] Asseng, S. , F. Ewert , C. Rosenzweig , et al. 2013. “Uncertainty in Simulating Wheat Yields Under Climate Change.” Nature Climate Change 3, no. 9: 827–832.

[fsn370035-bib-0005] Asseng, S. , F. Ewert , P. Martre , et al. 2015. “Rising Temperatures Reduce Global Wheat Production.” Nature Climate Change 5, no. 2: 143–147.

[fsn370035-bib-0007] Asseng, S. , P. Martre , A. Maiorano , et al. 2019. “Climate Change Impact and Adaptation for Wheat Protein.” Global Change Biology 25, no. 1: 155–173. 30549200 10.1111/gcb.14481

[fsn370035-bib-0008] Bencze, S. , O. Veisz , and Z. Bedo . 2004. “Effects of High Atmospheric CO2 and Heat Stress on Phytomass, Yield and Grain Quality of Winter Wheat.” Cereal Research Communications 32, no. 1: 75–82.

[fsn370035-bib-0009] Carter, B. P. , C. F. Morris , and J. A. Anderson . 1999. “Optimizing the SDS Sedimentation Test for End‐Use Quality Selection in a Soft White and Club Wheat Breeding Program.” Cereal Chemistry 76, no. 6: 907–911.

[fsn370035-bib-0010] Farooq, M. , H. Bramley , J. A. Palta , and K. H. Siddique . 2011. “Heat Stress in Wheat During Reproductive and Grain‐Filling Phases.” Critical Reviews in Plant Sciences 30, no. 6: 491–507.

[fsn370035-bib-0011] Ghaffari, A. , and M. Jalal Kamali . 2013. “Wheat Productivity in Islamic Republic of Iran: Constraints and Opportunities.” In Proceedings of the Regional Consultation on Improving Wheat Productivity in Asia, edited by R. Paroda , S. Dasgupta , B. Mal , S. S. Singh , M. L. Jat , and G. Singh , 98–111. CIMMYT.

[fsn370035-bib-0012] ICC Standard . 1998. “Determination of protein by Near Infrared Reflectance (NIR) spectroscopy. No. 159.”

[fsn370035-bib-0013] IPCC . 2018. “Global Warming of 1.5°C: An IPCC Special Report On The Impacts Of Global Warming of 1.5°C Above Pre‐Industrial Levels and Related Global Greenhouse Gas Emission Pathways, in the Context of Strengthening the Global Response to the Threat of Climate Change, Sustainable Development, and Efforts to Eradicate Poverty, Intergovernmental Panel on Climate Change 616 pp.”

[fsn370035-bib-0014] Irmak, S. , H. A. Naeem , G. L. Lookhart , and F. MacRitchie . 2008. “Effect of Heat Stress on Wheat Proteins During Kernel Development in Wheat Near‐Isogenic Lines Differing at Glu‐D1.” Journal of Cereal Science 48, no. 2: 513–516.

[fsn370035-bib-0015] Kino, R. I. , T. K. Pellny , R. A. Mitchell , A. Gonzalez‐Uriarte , and P. Tosi . 2020. “High Post‐Anthesis Temperature Effects on Bread Wheat ( *Triticum aestivum* L.) Grain Transcriptome During Early Grain‐Filling.” BMC Plant Biology 20: 1–17. 32299364 10.1186/s12870-020-02375-7 PMC7164299

[fsn370035-bib-0016] Koga, S. , U. Böcker , A. Moldestad , et al. 2016. “Influence of Temperature During Grain Filling on Gluten Viscoelastic Properties and Gluten Protein Composition.” Journal of the Science of Food and Agriculture 96, no. 1: 122–130. 25565275 10.1002/jsfa.7068

[fsn370035-bib-0017] Leghari, S. J. , N. A. Wahocho , G. M. Laghari , et al. 2016. “Role of Nitrogen for Plant Growth and Development: A Review.” Advances in Environmental Biology 10, no. 9: 209–219.

[fsn370035-bib-0018] Lobell, D. B. , W. Schlenker , and J. Costa‐Roberts . 2011. “Climate Trends and Global Crop Production Since 1980.” Science 333, no. 6042: 616–620. 21551030 10.1126/science.1204531

[fsn370035-bib-0019] Mutwali, N. I. , A. I. Mustafa , Y. S. Gorafi , and I. A. Mohamed Ahmed . 2016. “Effect of Environment and Genotypes on the Physicochemical Quality of the Grains of Newly Developed Wheat Inbred Lines.” Food Science & Nutrition 4, no. 4: 508–520. 27386101 10.1002/fsn3.313 PMC4930495

[fsn370035-bib-0020] Nuttall, J. G. , G. J. O'leary , J. F. Panozzo , C. K. Walker , K. M. Barlow , and G. J. Fitzgerald . 2017. “Models of Grain Quality in Wheat‐A Review.” Field Crops Research 202: 136–145.

[fsn370035-bib-0021] Oury, F. X. , and C. Godin . 2007. “Yield and Grain Protein Concentration in Bread Wheat: How to Use the Negative Relationship Between the Two Characters to Identify Favourable Genotypes?” Euphytica 157, no. 1: 45–57.

[fsn370035-bib-0022] Pinto, R. S. , M. P. Reynolds , K. L. Mathews , C. L. McIntyre , J. J. Olivares‐Villegas , and S. C. Chapman . 2010. “Heat and Drought Adaptive QTL in a Wheat Population Designed to Minimize Confounding Agronomic Effects.” Theoretical and Applied Genetics 121: 1001–1021. 20523964 10.1007/s00122-010-1351-4 PMC2938441

[fsn370035-bib-0035] Qiao, S. , S. P. Harrison , I. C. Prentice , et al. 2023. “Optimality‐based modelling of wheat sowing dates globally.” Agricultural Systems 206: 103608. https://www.sciencedirect.com/science/article/pii/S0308521X23000136.

[fsn370035-bib-0023] R Core Team . 2021. “R: A Language and Environment for Statistical Computing.” R Foundation for Statistical Computing, Vienna, Austria. https://www.R‐project.org/.

[fsn370035-bib-0024] Semenov, M. A. , and N. G. Halford . 2009. “Identifying Target Traits and Molecular Mechanisms for Wheat Breeding Under a Changing Climate.” Journal of Experimental Botany 60, no. 10: 2791–2804. 19487387 10.1093/jxb/erp164

[fsn370035-bib-0026] Shewry, P. R. , and A. S. Tatham . 1997. “Disulphide Bonds in Wheat Gluten Proteins.” Journal of Cereal Science 25, no. 3: 207–227.

[fsn370035-bib-0025] Shewry, P. R. , and S. J. Hey . 2015. “The Contribution of Wheat to Human Diet and Health.” Food and Energy Security 4, no. 3: 178–202. 27610232 10.1002/fes3.64 PMC4998136

[fsn370035-bib-0027] Skylas, D. J. , D. Van Dyk , and C. W. Wrigley . 2005. “Proteomics of Wheat Grain.” Journal of Cereal Science 41, no. 2: 165–179.

[fsn370035-bib-0028] Ullah, A. , F. Nadeem , A. Nawaz , K. H. Siddique , and M. Farooq . 2022. “Heat Stress Effects on the Reproductive Physiology and Yield of Wheat.” Journal of Agronomy and Crop Science 208, no. 1: 1–17.

[fsn370035-bib-0029] Wang, Q. , F. Jia , X. Zhang , X. Wang , J. Li , and J. Wang . 2020. “Transcriptome Analysis Reveals That the Multiple Metabolic Pathways Were Related to Gluten Polymerization in Different Quality Wheats (*Triticum aestivum* L.).” Food Science & Nutrition 8, no. 8: 4573–4583. 32884737 10.1002/fsn3.1769 PMC7455946

[fsn370035-bib-0030] Yan, W. 2001. “GGEbiplot—A Windows Application for Graphical Analysis of Multienvironment Trial Data and Other Types of Two‐Way Data.” Agronomy Journal 93: 1111–1118.

[fsn370035-bib-0031] Zadoks, J. C. , T. T. Chang , and C. F. Konzak . 1974. “A Decimal Code for the Growth Stages of Cereals.” Weed Research 14, no. 6: 415–421.

[fsn370035-bib-0032] Zampieri, M. , A. Ceglar , F. Dentener , and A. Toreti . 2017. “Wheat Yield Loss Attributable to Heat Waves, Drought and Water Excess at the Global, National and Subnational Scales.” Environmental Research Letters 12, no. 6: 64008.

[fsn370035-bib-0033] Zhang, X. , S. Chen , H. Sun , Y. Wang , and L. Shao . 2012. “Effects of High Temperature on Grain Growth and Starch Accumulation in Wheat Under Field Conditions.” Field Crops Research 138: 33–40.

[fsn370035-bib-0034] Zhong, Y. , Q. Zhou , and D. Jiang . 2022. “Wheat Quality Under Global Climate Change: Consequences, Mechanisms, and Countermeasures.” In Sustainable Crop Productivity and Quality Under Climate Change, 103–135. Elsevier.

